# Conductivity of Nanowire Arrays under Random and Ordered Orientation Configurations

**DOI:** 10.1038/srep10219

**Published:** 2015-05-15

**Authors:** Milind Jagota, Nelson Tansu

**Affiliations:** 1 Center for Photonics and Nanoelectronics, Department of Electrical and Computer Engineering, Lehigh University, Bethlehem, PA 18015, USA

## Abstract

A computational model was developed to analyze electrical conductivity of random metal nanowire networks. It was demonstrated for the first time through use of this model that a performance gain in random metal nanowire networks can be achieved by slightly restricting nanowire orientation. It was furthermore shown that heavily ordered configurations do not outperform configurations with some degree of randomness; randomness in the case of metal nanowire orientations acts to increase conductivity.

Transparent conductors are needed widely for flat screen displays, touch screens, solar cells, and light-emitting diodes, among many other technologies 1,2,3,4,5,6,7,8,9. Indium Tin Oxide (ITO) is currently the most widely used material for transparent conductors due to its high conductivity and high transparency 10. The ITO based technology has several issues, however; its scarcity drives up its price, it requires an expensive sputter deposition manufacturing process, and it is brittle, a particularly undesirable characteristic for modern flexible electronics. Of the several replacements for ITO currently being researched, random networks of metal nanowires show the most potential to match ITO in both transparency and conductivity. In addition, the metal nanowire-based technologies display better flexibility and are more compatible with manufacturing processes than ITO films 2,3,11. Random networks made of silver nanowires, specifically, have entered the market in their first forms 12. This technology, however, is still in an early phase of development and can undoubtedly be improved in performance.

Much of recent research into random silver nanowire networks has focused on development of computational models to investigate dependence of conductivity of networks on a variety of different factors. Specifically, the effects of rod orientation on conductivity of networks have shown promise as a possible way to improve performance of metal nanowire networks, for applications where current need only flow in one direction 13,14,15,16. Du *et al.* 15 experimentally studied the effects of orientation on conductivity of 3D carbon nanotube composites, finding that a slight degree of alignment improves conductivity. They also used a computational model to study how percolation probability of 2D random rod dispersions is affected by rod orientation. This study, however, did not include results of conductivity of 2D rod dispersions. White *et al.* 16, in a follow up paper to 15, developed a more sophisticated computational model capable of calculating conductivity of 3D rod dispersions, again finding that a slight degree of axial alignment improves conductivity. Behnam *et al.* 13,14 utilized simulations to analyze the dependence of resistivity of random carbon nanotube films on nanotube density, nanotube length, and nanotube alignment, among other factors. More recently, Mutiso *et al.* 17 adapted the conductivity model developed in 16 for 2D metal nanowire networks. They used the model to extract contact resistance in silver nanowire systems and find dependence of conductivity on area fraction of nanowires.

In this work, we demonstrate our own computational model for simulation of metal nanowire networks, which exhibits good fit with experimental results and previously published computational results. We then use this model to extract results for the first time on how conductivity of random metal nanowire networks is affected by different orientation restrictions of varying randomness. Two different orientation configurations are reported. In the first, a uniform distribution of orientations over the range (*−θ*, *θ*) with respect to a horizontal line is used. In the second, a distribution of orientations over the range [*−θ*] ∪ [*θ*] is used, also with respect to a horizontal line. In each case *θ* is gradually decreased from 90° to 0°. Conductivity is measured both in directions parallel and perpendicular to alignment. It was found that a significant improvement in conductivity parallel to direction of alignment can be obtained by slightly restricting orientation of the uniform distribution. This improvement, however, comes at the expense of a larger drop in perpendicular conductivity. The general form of these results matches that demonstrated by Behnam *et al.* 13 for carbon nanotube films, although specific values differ. Surprisingly, it was found that the highly ordered second case is unable to outperform isotropic networks for any value of *θ*; this demonstrates that continuous orientation configurations with some degree of randomness are preferable to highly ordered configurations.

The computational model employed in this study is structured similarly to those previously demonstrated by Mutiso *et al.* 17 and Behnam *et al.* 13,14. Random networks of nanowires are modeled by a network of randomly generated line segments. These nanowires are then assembled into a resistor circuit matrix using Kirchoff’s junction rule, where each length of wire and each junction between two wires is a resistor. A voltage arbitrarily set to 10 V is then applied from left to right across the network. Figure 1 shows two examples of these networks at different concentrations; fig. 1(a) is at a length concentration just above the critical concentration of percolation, *C*_*p*_, whereas fig. 1(b) is at a length concentration far above *C*_*p*_. The junction resistance is assumed to be a constant value *R*_*J*_; the validity of this assumption was demonstrated by Mutiso *et al.* 17. The resistance of the wires, *R*_*rod*_, is calculated using the resistivity of silver, and values for the length and diameter of the rods. The length of the nanowires is set to 35 μm with diameter of 65 nm. This geometry is chosen based on silver nanowires which are commercially available from Seashell Technologies^™^ 18.

A matrix equation is then set up and solved to find the voltage at each junction, which can be used to calculate conductivity of the network. Figure 2 is a 2-D color map of voltage at each node of an array with concentration much above critical percolation concentration (*C* *~* *5 C*_*p*_ ). It shows that voltage uniformly decreases across a high concentration network as expected. We include *R*_*rod*_ in our calculations despite the fact that it is on the order of 10^−2^ times smaller than *R*_*J*_ so that the model may be applied later to similar situations where *R*_*rod*_ may be comparable to *R*_*J*_.

Figure 3 shows conductivity as a function of length concentration for isotropic samples. The conductivity, *k*, is the sheet conductivity of the samples. Since junction resistance (*R*_*J*_) is dominant in this system, we define a normalized conductivity *k*_*N*_ as:


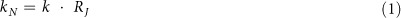


The length concentration, *C*, is defined as the number density of rods multiplied by the length of each rod. We define a normalized concentration *C*_*N*_ as:


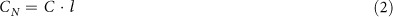


where *l* is the length of each rod. We choose to normalize in this way because *C*_*N*_ is independent of rod parameters. For example, the critical percolation concentration *C*_*p*_ for rods in 2D space can be determined using the following formula 3:


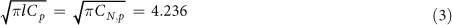


Normalizing *C*_*p*_ by multiplying by *l* thus yields a value of 5.71 which is constant for any set of rod parameters. This value is marked on Fig. 3. Since our manipulated variable is independent of rod parameters, it is necessary to conduct simulations only with one rod length. The inset diagram in fig. 3 is a log-log plot of normalized concentration as a function of *C*_*N*_  − *C*_*N,p*_. This plot is a straight line, which suggests a power law dependence of conductivity on *C*_*N*_ − *C*_*N,p*_, of the form:


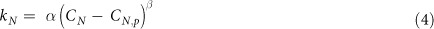


This equation (4) is the accepted general form of dependency of conductivity on concentration 19. A best line fit, fitted only through values of *C*_*N*_  − *C*_*N,p*_ greater than 7 to avoid the heavy fluctuations of lower values, indicates the critical exponent *β* = 1.73. This agrees well with the accepted value of *β* for a junction resistance dominated network of 1.75 17. The agreement with previously published results validates our model for random metal nanowire networks.

Figure 4(a) shows normalized conductivity, defined in the same way as in fig. 3, as a function of *θ*, where orientation of the rods is restricted over (*−θ*, *θ*) from a horizontal line parallel to the boundary of the space. We refer to this horizontal line as *L*_*h*_. Fig. 4(b) shows an example of one such sample with *θ* = 45°. It is important to note that when orientation is restricted in this way, the networks are no longer rotationally symmetrical; conductivity measured in the same direction as *L*_*h*_ is different from conductivity measured perpendicular to *L*_*h*_. In this sample, conductivity in the same direction as *L*_*h*_ is the conductivity obtained by applying a potential difference from the left of the sample to the right (or vice-versa). Conductivity perpendicular to *L*_*h*_ is the conductivity obtained by applying a potential difference from the top of the sample to the bottom (or vice-versa). All samples are generated at a normalized concentration of 29, with all other variables the same as in fig. 3. It can be seen that by reducing *θ* from 90°, the isotropic state, conductivity perpendicular to *L*_*h*_ immediately begins to decrease. Conductivity in the same direction as *L*_*h*_, however, increases slightly before decreasing, reaching a maximum around *θ* = 60°. The improvement in conductivity over the isotropic state is an approximately 20% gain in conductivity. This is a significant improvement in performance for applications which only require current flow in one direction.

This pair of effects can be described as the result of two competing effects that result from decreasing *θ*. As *θ* is reduced, the number of intersections and number of parallel paths decreases, reducing conductivity. At the same time, the number of junctions the current must travel through decreases (same direction as *L*_*h*_) or increases (perpendicular to *L*_*h*_). This increases conductivity in the same direction as *L*_*h*_, and further decreases conductivity perpendicular to *L*_*h*_. In the same direction as *L*_*h*_, these effects are competing. This allows conductivity to initially increase before decreasing when the lack of parallel paths dominates. In the direction perpendicular to *L*_*h*_, both effects act to decrease conductivity, causing conductivity to immediately drop.

Figure 5(a) shows normalized conductivity as a function of *θ*, where rod orientation is restricted in the range [*−θ*] ∪ [*θ*] from the same horizontal line *L*_*h*_. All other variables are held constant with fig. 4. Fig. 5(b) shows an example of one such sample, with *θ* = 45°. Note that for any value of *θ* taken with respect to *L*_*h*_, the value 90° − *θ* is exactly equivalent when taken with respect to a line perpendicular to *L*_*h*_. For the case where *θ* = 45°, as in fig. 5(b), it does not matter whether conductivity is measured along or perpendicular to *L*_*h*_. For any other value of θ with conductivity measured along *L*_*h*_, an equivalent network is obtained by taking 90° − *θ* and measuring conductivity perpendicular to *L*_*h*._

Even though the configuration shown in fig. 5 is much more controlled than the restricted random distribution shown in fig. 4, no performance gain over an isotropic sample is achieved for any value of *θ*. *θ* = 30° is equivalent in conductivity to an isotropic sample; any other value of *θ* causes a decrease in conductivity. This yields the unexpected result that highly ordered orientation configurations do not yield the greatest increases in conductivity. In fact, randomness in this case acts to improve conductivity, and should not be removed.

In summary, metal nanowire networks show great potential for application in various forms of technology. Our computational model, which has proven itself accurate through its good fit with previously published data, has demonstrated quantitatively how different orientation configurations can impact conductivity of metal nanowire networks. Restriction of orientation can improve conductivity in a single direction by significant amounts; this could be relevant in a variety of technologies where current flow is only required in one direction. Surprisingly, heavily controlled orientation configurations do not exhibit superior conductivity; some degree of randomness in orientation in fact acts to improve conductivity of the networks.

## Author Contributions

M. J. and N. T. contributed to the discussions, concept development, theoretical analysis, analysis of the results, and writing of the manuscript. M. J. wrote the simulation program used in this study, and N. T. supervised the studies performed in the manuscript. Both authors reviewed and made the final editing in the manuscript.

## Additional Information

**How to cite this article**: Jagota, M. and Tansu, N. Conductivity of Nanowire Arrays under Random and Ordered Orientation Configurations. *Sci. Rep.* **5**, 10219; doi: 10.1038/srep10219 (2015).

## Figures and Tables

**Figure 1:**
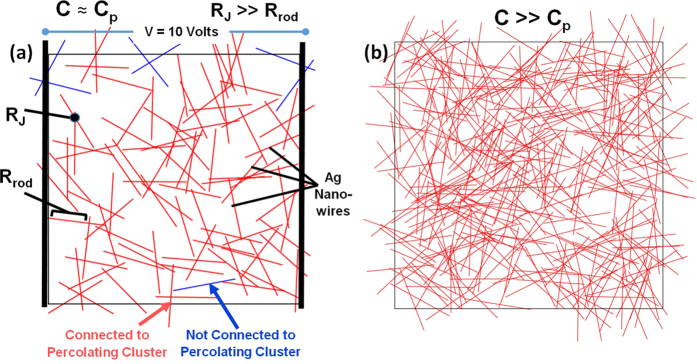
Sample networks of Ag nanowires for 140 μm × 140 μm domain with (**a**) concentration just above critical percolation concentration (C ~ C_p_), and (**b**) concentration much above critical percolation concentration (C»C_p_). The voltage is applied across the horizontal direction across the domain length.

**Figure 2:**
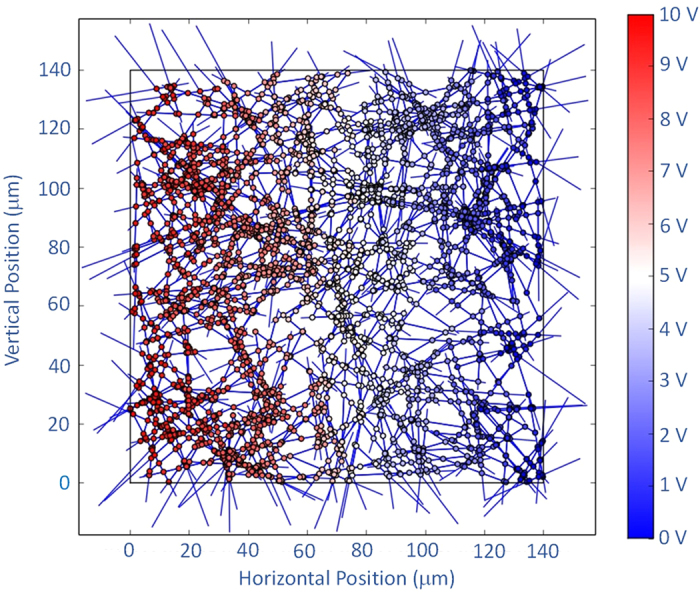
2-D color map showing voltage at different nodes in computational domain for network arrays with concentration much higher than the percolation concentration (C ~ 5 C_p_). The dots represent voltages at each nodes. Left border voltage is 10 V, right border voltage is 0 V.

**Figure 3:**
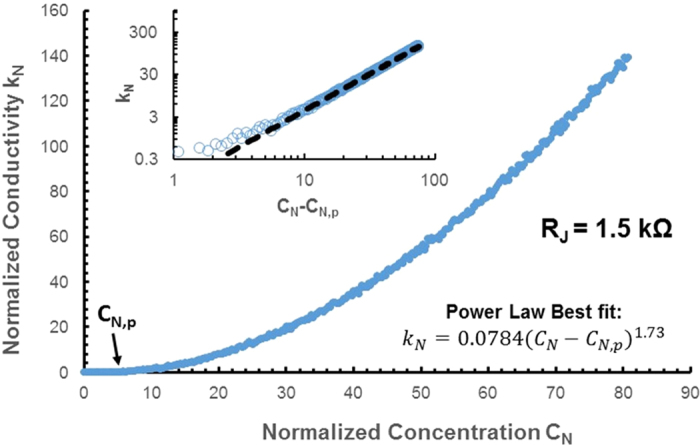
The main plot shows the relation of normalized conductivity (k_N_) versus normalized length concentration (C_N_) for the regime above and below normalized percolation concentration (C_N,p_). The inset shows the relation of the normalized conductivity (k_N_) vs normalized concentration minus critical concentration (C_N_ − C_N,p_) in logarithmic scale. The relation of the k_N_ and (C_N_ − C_N,p_) is also presented by using power law fitting relation.

**Figure 4:**
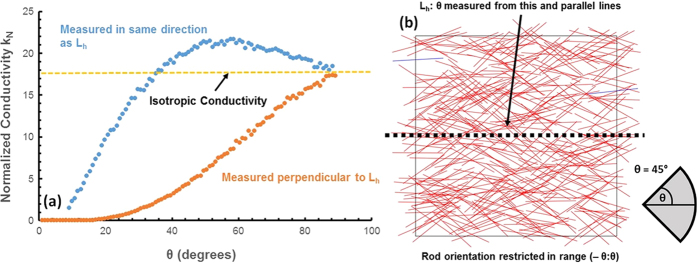
Data showing (**a**) the relation between normalized conductivity (k_N_) and θ at C_N_ = 29, where rod orientation is distributed uniformly over (−θ, θ) with respect to L_h_, measured along and perpendicular to L_h_, and (**b**) a sample network using uniform orientation distribution of (−θ, θ) of size 140 μm × 140 μm at C_N_ = 29 where θ = 45°.

**Figure 5:**
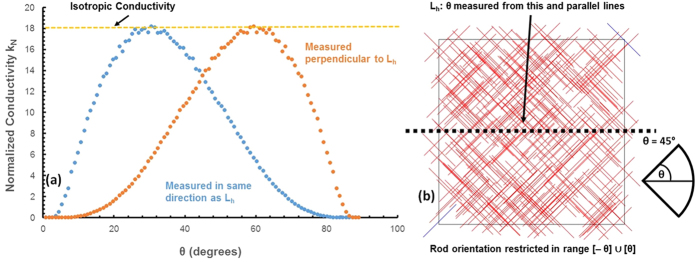
Data showing (**a**) the relation between normalized conductivity (k_N_) and θ at C_N_ = 29, where rod orientation is distributed over [−θ] ∪ [θ] with respect to L_h_, measured along and perpendicular to L_h_, and (**b**) a sample network using orientation distribution over [−θ] ∪ [θ] of size 140 μm × 140 μm at C_N_ = 29 where θ = 45°.

## References

[b1] De S. & Coleman J. N. The effects of percolation in nanostructured transparent conductors. MRS Bulletin, 36, 774 (2011).

[b2] Hecht D. S., Hu L. & Irvin G. Emerging Transparent Electrodes Based on Thin Films of Carbon Nanotubes, Graphene, and Metallic Nanostructures. Advanced Materials, 23, 1482 (2011). 21322065 10.1002/adma.201003188

[b3] Hu L., Wu H. & Cui Y. Metal nanogrids, nanowires, and nanofibers for transparent electrodes. MRS Bulletin, 36, 760 (2011).

[b4] Kumar A. & Zhou C. The Race To Replace Tin-Doped Indium Oxide: Which Material Will Win? ACS Nano, 4, 11 (2010). 20099909 10.1021/nn901903b

[b5] Dahal R., Pantha B., Li J., Lin J. Y. & Jiang H. X. InGaN/GaN multiple quantum well solar cells with long operating wavelengths. Appl. Phys. Lett., 94, 063505 (2009).

[b6] Arif R. A., Ee Y. K. & Tansu N. Polarization engineering via staggered InGaN quantum wells for radiative efficiency enhancement of light emitting diodes. Appl. Phys. Lett., 91, 091110 (2007).

[b7] Koo W. H., *et al.* Light Extraction of Organic Light Emitting Diodes by Defective Hexagonal-Close-Packed Array. Advanced Functional Materials, 22, 3454 (2012).

[b8] Tan C. K. & Tansu N. Nanostructured Lasers: Electrons and Holes Get Closer. Nature Nanotechnology, 10, 107 (2015). 10.1038/nnano.2014.333 25599192

[b9] Malyutenko V. K., Bolgov S. S. & Podoltsev A. D. Current crowding effect on the ideality factor and efficiency droop in blue lateral InGaN/GaN light emitting diodes. Appl. Phys. Lett., 97, 251110 (2010).

[b10] Gordon R. G. Criteria for Choosing Transparent Conductors. MRS Bulletin, 25, 52 (2000).

[b11] Lee J. Y., Connor S. T., Cui Y. & Peumans P. Solution-processed metal nanowire mesh transparent electrodes. Nano Letters 8, 689 (2008). 18189445 10.1021/nl073296g

[b12] http://www.cambrios.com/technology (ClearOhm^®^ Silver Nanowire Coating Material, accessed on February 2015).

[b13] Behnam A., Guo J. & Ural A. Effects of nanotube alignment and measurement direction on percolation resistivity in single-walled carbon nanotube films. Journal of Applied Physics, 102, 044313 (2007).

[b14] Behnam A. & Ural A. Computational study of geometry-dependent resistivity scaling in single-walled carbon nanotube films. Physical Review B, 75, 125432 (2007).

[b15] Du F., Fischer J. E. & Winey K. I. Effect of nanotube alignment on percolation conductivity in carbon nanotube/polymer composites. Physical Review B, 72, 121404 (2005).

[b16] White S. I., DiDonna B. A., Mu M., Lubensky T. C. & Winey K. I. Simulations and electrical conductivity of percolated networks of finite rods with various degrees of axial alignment. Physical Review B, 79, 024301 (2009).

[b17] Mutiso R. M., Sherrott M. C., Rathmell A. R., Wiley B. J. & Winey K. I. Integrating Simulations and Experiments To Predict Sheet Resistance and Optical Transmittance in Nanowire Films for Transparent Conductors. ACS Nano, 7, 7654 (2013). 23930701 10.1021/nn403324t

[b18] http://www.seashelltech.com/ (Seashell Technology, accessed on February 2015).

[b19] Stauffer D. & Aharony A. Introduction to percolation theory. CRC Press 1994.

